# Hemographic indices are associated with mortality in acute heart failure

**DOI:** 10.1038/s41598-017-17754-8

**Published:** 2017-12-19

**Authors:** Wei-Ming Huang, Hao-Min Cheng, Chi-Jung Huang, Chao-Yu Guo, Dai-Yin Lu, Ching-Wei Lee, Pai-Feng Hsu, Wen-Chung Yu, Chen-Huan Chen, Shih-Hsien Sung

**Affiliations:** 1 Department of Medicine, Taipei Veterans General Hospital, Taipei, Taiwan; 2 Department of Medical Education, Taipei Veterans General Hospital, Taipei, Taiwan; 3 Department of Medicine, National Yang-Ming University, Taipei, Taiwan; 4 Department of Public Health, National Yang-Ming University, Taipei, Taiwan

## Abstract

Hemographic indices have been associated with clinical outcomes in patients with chronic heart failure. We therefore investigated the prognostic values of hemographic indices in patients hospitalized for acute heart failure (AHF). Patients hospitalized primarily for AHF were drawn from an intramural registry. Hemographic indices, including white blood cell counts, neutrophil counts, neutrophil-to-lymphocyte ratio, reciprocal of lymphocyte (RL) and platelet-to-lymphocyte ratio were recorded. Among a total of 1923 participants (mean age 76 ± 12 years, 68% men), 875 patients died during a mean follow-up of 28.6 ± 20.7 months. Except for white blood cell counts, all the other hemographic indices were related to mortality, independently. In a forward stepwise Cox regression analysis among hemographic indices, RL was the strongest predictor (HR and 95% CI per-1SD:1.166,1.097–1.240) for mortality, after accounting for confounders. However, conditioned on the survivals, the hemographic indices were independently related to mortality within 3 years of follow-up, rather than beyond. Hemographic indices were independent risk factors of mortality in patients hospitalized for AHF, especially in patients with impaired left ventricular systolic function. As an acute presentation of inflammation, hemographic indices might be useful to identify subjects at risk of mortality soon after the index hospitalization.

## Introduction

Hemographic indices, measures related to white blood cells, are simple and important indicators of not only systemic infection but also subclinical inflammation^1,2^. While leukocytosis, especially neutrocytosis plus lymphocytopenia, as well as thrombocytosis are representatives of active inflammation, observational studies have shown their prognostic impacts in patients with autoimmune disease, malignancy, stable coronary artery disease (CAD), and acute coronary syndrome (ACS), respectively^3–7^. In subjects with chronic heart failure (CHF), a slight but relevant subclinical inflammation was observed, in comparison to the controls^8,9^. Given the inflammation was associated with structural and functional remodeling of left ventricle^10^, it has been suggested that inflammation may involve the pathogenesis of CHF and influence the clinical outcomes^2,11,12^.

In patients with acute heart failure (AHF), Uthamalingam *et al*. have shown that the neutrophil-to-lymphocyte ratio (NLR) rather than neutrophil count per se was independently related to the in-hospital mortality and post-discharge clinical outcomes, regardless of the left ventricular function^13^. In contrast, Polat *et al*. demonstrated decreased platelet and lymphocyte counts were independently correlated with one-year mortality in 119 hospitalized subjects with heart failure and reduced ejection fraction (HFrEF)^14^. However, the associated risks of various hemographic indices haven’t been comprehensively compared in subjects with AHF. Neither the divergence regarding clinical impacts of hemographic indices on long-term survival in patients with HFrEF or preserved ejection fraction (HFpEF) was studied. We therefore investigated the prognostic values of hemographic indices, including total white blood cell (WBC) count, neutrophil count, reciprocal of lymphocyte (RL), neutrophil-to-lymphocyte ratio (NLR), and platelet-to-lymphocyte ratio (PLR) in patients hospitalized for AHF.

## Methods

### Study population

The study population was drawn from the HARVEST registry (Heart Failure Registry of Taipei Veterans General Hospital), which was composed by patients hospitalized for AHF from October 2003 to December 2012^15^. Patients with severe infection, ACS, severe hepatic disease, hematopoietic diseases, and active malignancy were excluded. A total of 2663 patients, who have received echocardiographic studies before discharge, were eligible. The investigation was conformed to the principles outlined in the Declaration of Helsinki, and was approved by Institutional Review Board Taipei Veterans General Hospital (IRB-VGHTPE). As a retrospective cohort data, patient informed consent was waived by IRB-VGHTPE.

Data of demographic characteristics, hemogram, biochemistry, and echocardiographic features were collected during the period of hospitalization in a web-based electronic medical recording system. The prescribed medications at discharge were also recorded. Renin-angiotensin system (RAS) blockades were referred to angiotensin-converting enzyme inhibitors and angiotensin II receptor blockers.

### Definitions of Hemographic indices

The hemograms of the study population were obtained at the first presentation of the patients in the hospital. WBC count, neutrophil count, RL, NLR, and PLR were referred to be the hemographic indices. NLR and PLR were calculated as the ratios of the neutrophil or platelet counts to the lymphocyte count, respectively. RL was the reciprocal of lymphocyte percentage in white blood cells.

### Laboratory Data and Echocardiography

The left ventricular ejection fraction (LVEF) was derived from the 2D-guided M-mode echocardiography^16^. E/A was the ratio of left ventricular early (E) to late (A) filling flow velocity, and septal E/e′ represented the ratio of early ventricular filling flow velocity (E) to the septal mitral annulus tissue velocity (e′). HFrEF was defined as subjects with a LVEF < 50%^17^. According to modified glomerular filtration rate estimating equation for Chinese patients, estimated glomerular filtration (eGFR) rate was calculated^18^. The stage of chronic kidney disease (CKD) was based on KDOQI guidelines^19^. The commercialized measure for N-terminal pro-brain natriuretic peptide (NT-proBNP; Roche Diagnostics, Basel, Switzerland) was available for patients hospitalized after 2009.

### Follow-up

The causes and dates of death for the study population who had deceased before December, 2012 were obtained by linking our database with the National Death Registry through a unique, personal identification number given to every Taiwan citizen. The International Classification of Disease, Ninth Revision (ICD-9) of 390 to 459 was classified as cardiovascular death^20^.

### Statistical analysis

Nonnormally distributed continuous variables were presented as geometric means and standard deviation and normally distributed continuous variables were reported as mean ± standard deviation. Categorical variables were expressed as the absolute numbers and relative frequencies. Baseline characteristics were compared by Student’s t-test or Chi-square tests as appropriate. The prognostic differences across the tertiles of hemographic indices were analyzed by Kaplan-Meier survival analysis. Cox proportional hazards models were used to evaluate the independence of hemographic indices in the prediction of mortality with adjustments for age, sex, mean blood pressure, LVEF, hemoglobin, eGFR, sodium, and prescribed medications. Because of skewed distribution, WBC count, neutrophil count, and NT-proBNP levels were taken log transformation for Cox regression analyses. Forward stepwise multiple logistic regression analyses were used to compare the predictive values between hemographic indices, after accounting for age, sex, mean blood pressure, LVEF, hemoglobin, eGFR, sodium, and prescribed medications. Collinearity in the multivariale Cox regression models was examined by calculating the variance inflation factor. No significant collinearity was found in any of the Cox regression models. In order to analyze whether the prognostic impacts of hemographic indices abated along the follow-up period, we conducted Cox regression analyses with adjustments for age and gender at different time slots of within 1 year, between 1 to 3 years, and between 3 to 5 years conditioned on the survivals. The stratified incidence rate and rate ratio were calculated and compared by using a generalized linear model with Poisson distribution. All the statistical analyses were performed SPSS v.16.0 software (SPSS, Inc., Chicago, IL, USA). All the tests performed were two-sided and a P value < 0.05 was considered statistically significant.

## Results

Among a total of 1923 patients (age 76 ± 12 years, 68% men, 21% de novo heart failure) in this analysis, 875 patients died during a mean follow-up duration of 28.6 ± 20.7 months. Table 1 discloses the baseline characteristics between the dead and who survived. In short, the dead were older, less likely to have hypertension, had lower mean blood pressure and LVEF, lower levels of hemoglobin and eGFR, and higher NT-proBNP levels. Sex distribution and presence of diabetes, CAD, atrial fibrillation and stroke were similar in both groups. Left ventricular mass, E/A and E/e′ were not different between the groups. Comparing to the survivors, WBC count, neutrophil count, PLR, NLR and RL were higher, but lymphocyte count and hemoglobin level were lower in the dead. In addition, the prescription rates of RAS blockades, beta-blockers, and spironolactone were higher in the survivors than the dead.

**Table 1:** Baseline characteristics of the study population.

	Survived, n = 1048	Mortality, n = 875	P value
*Age (years)*	74.8 ± 13.9	78.3 ± 10.7	<0.01
*Male gender, n (%)*	697(66.6)	610(69.7)	0.14
*Mean BP (mmHg)*	103.1 ± 21.9	99.0 ± 21.6	<0.01
*De novo heart failure, n (%)*	229 (21.9)	175 (20.0)	0.34
*Heart failure with reduced EF*	381(36.5)	379(43.3)	<0.01
***Co-morbidity, n (%)***			
Hypertension	651(62.1)	501(57.3)	0.03
Diabetes mellitus	384(36.6)	339(38.7)	0.34
Coronary artery disease	364(34.7)	314(35.9)	0.60
Atrial fibrillation	321(30.6)	244(27.9)	0.19
Stroke	96(9.2)	74(8.5)	0.59
***Echocardiography***			
LVEF (%)	55.4 ± 20.0	53.4 ± 20.7	0.03
LV mass (gm)	282.9 ± 113.5	284.0 ± 105.9	0.82
E/A ratio	1.05 ± 0.66	1.15 ± 0.82	0.10
Septal E/E’	17.4 ± 8.0	18.3 ± 7.8	0.16
***Hemogram***			
*WBC count (/mm^3^)	6924.68 ± 1.41	7156.48 ± 1.47	0.051
*Neutrophil count (/mm^3^)	4602.56 ± 1.57	5006.10 ± 1.64	<0.01
*Lymphocyte count (/mm^3^)	1269.69 ± 1.63	1077.20 ± 1.68	<0.01
Reciprocal of Lymphocyte	6.45 ± 5.63	8.07 ± 7.07	<0.01
Neutrophil-to-lymphocyte ratio	4.76 ± 5.35	6.26 ± 6.80	<0.01
Platelet-to-lymphocyte ratio (k/mm^3^)	173.1 ± 120.9	207.0 ± 246.7	<0.01
Hemoglobin (g/dl)	12.13 ± 2.33	11.57 ± 2.11	<0.01
Platelet count (k/mm^3^)	203.8 ± 92.8	201.4 ± 92.1	0.57
***Biochemistry***			
Creatinine (mg/dL)	1.78 ± 1.48	1.92 ± 1.27	0.03
eGFR (ml/min/1.73 m^2^)	56.0 ± 30.4	48.3 ± 27.6	<0.01
Sodium (mEq/L)	138.9 ± 4.2	138.6 ± 5.3	0.15
Potassium (mEq/L)	4.10 ± 0.73	4.11 ± 0.71	0.78
*NT-proBNP (pg/ml), n = 646	4818.3 ± 3.6	8395.1 ± 3.5	<0.01
***Medications, n (%)***			
Antiplatelet	715(71.7)	623(71.2)	0.82
Beta-blocker	727(69.4)	494(56.5)	<0.01
RAS blockade	905(86.4)	699(79.9)	<0.01
Spironolactone	628(59.9)	467(53.4)	<0.01

*Geometric means and standard deviation.

E/A ratio: ratio of the early (E) to late (A) ventricular filling velocities; E/e′: ratio of early ventricular filling velocity (E) to early diastolic tissue velocity mitral annulus; EF: ejection fraction; eGFR: estimated glomerular filtration rate; LV: left ventricular; NT-proBNP: N-terminal pro-brain natriuretic peptide; RAS blockade: renin-angiotensin system blockade; WBC: white blood cells

### Predictors of Mortality

**Figure 1:**
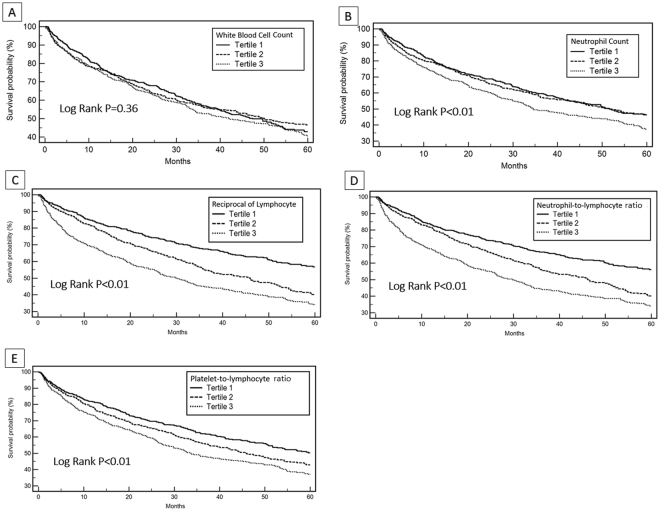
The Kaplan–Meier survival curve analysis of the study population, according to the tertiles of the levels of white blood cell count (**A**), neutrophil count (**B**), reciprocal of lymphocyte count (**C**), neutrophil-to-lymphocyte ratio (**D**), and platelet-to-lymphocyte ratio (**E**) in the study population.

In uni-variable Cox analysis, age, mean blood pressure, LVEF, hemoglobin, eGFR, sodium, and NT-proBNP levels were all related to long-term survival in the study population. (Supplementary Table S1) In addition, among the hemographic indices, neutrophil count, PLR, NLR and RL but not WBC count, were also positively associated with higher mortality during the follow-up. (Table 2, Model 1) The Kaplan-Meier survival curve analyses showed a decreasing survival probability along the tertile distributions of the hemographic indices, except for WBC count, in a 5-year follow-up duration. (Fig. 1) Furthermore, the prescriptions of RAS blockades, beta-blockers, and spironolactone significantly reduced mortality of the study population. (Supplementary Table S1)

**Table 2:** Predictors of 5-year mortality identified by uni- and multi-variable Cox regression analysis.

	**Model 1**		**Model 2**		**Model 3**	
	**Hazard ratio (95% CI)**	**P**	**Hazard ratio (95% CI)**	P	**Hazard ratio (95% CI)**	**P**
*While blood cell count, 1 SD = 1.44/mm^3^	1.055 (0.985–1.130)	0.12	—	—	—	—
*Neutrophil count, 1 SD = 1.60 /mm^3^	1.141 (1.066–1.222)	<0.01	1.133 (1.041–1.234)	<0.01	1.189 (1.023–1.383)	0.02
Reciprocal of Lymphocyte, 1 SD = 6.3	1.162 (1.115–1.211)	<0.01	1.165 (1.096–1.239)	<0.01	1.141 (1.017–1.280)	0.03
Neutrophil-to-lymphocyte ratio, 1 SD = 6.1	1.160 (1.112–1.210)	<0.01	1.162 (1.094–1.235)	<0.01	1.137 (1.015–1.274)	0.03
Platelet-to-lymphocyte ratio, 1 SD = 189.5 k/mm^3^	1.090 (1.052–1.129)	<0.01	1.161 (1.041–1.295)	<0.01	1.244 (1.033–1.498)	0.02

Model 1: crude ratio.

Model 2: Adjust age, sex, mean blood pressure, left ventricular ejection fraction, sodium and hemoglobin levels, estimated glomerular filtration rate, and use of renin-angiotensin system blockade, beta-blockade and spironolactone.

Model 3: Adjust variables in Model 1 **PLUS** N-terminal pro-brain natriuretic peptide (NT-proBNP).

*Log transformation of while blood cell count and neutrophil count.

In multi-variable Cox proportional hazard models, WBC count, neutrophil count, RL, NLR and PLR were all independent predictors of 5-year mortality after accounting for age, sex, mean blood pressure, LVEF, hemoglobin, eGFR, sodium, and prescribed medications. (Table 2, Model 2) With further adjustments for NT-proBNP, the neutrophil count, RL, NLR and PLR, but not white blood cell count remained significantly associated with long-term survival. (Table 2, Model 3) With fixed adjustments for age, gender, mean blood pressure, LVEF, hemoglobin, eGFR, sodium, and prescribed medications in a forward stepwise Cox regression analysis among hemographic indices, RL was the strongest predictor getting into the model [hazard ratios and 95% confidence interval: 1.165 (1.096–1.239)].

### Subgroup analysis

**Figure 2:**
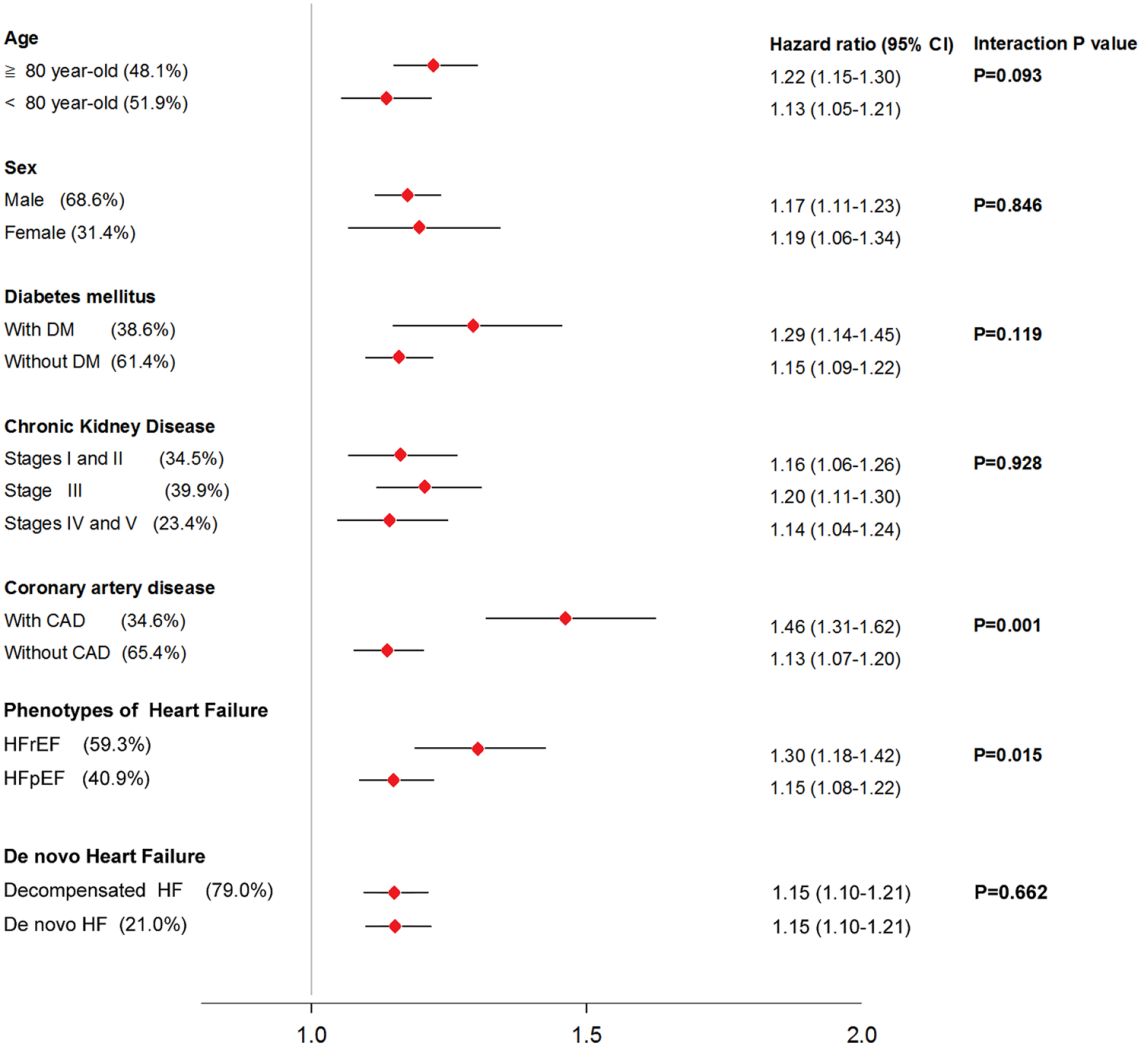
Hazard ratio (HR) and 95% confidence interval (CI) of per-1SD increase of reciprocal of lymphocyte count for mortality, after accounting for age and sex, were demonstrated in subgroups of age, gender, diabetes, coronary artery disease, chronic kidney disease, phenotypes of heart failure and de novo heart failure.

In subgroup analysis, RL was a significant predictor of long-term mortality across all the subpopulations, including subjects ≥ or < 80 years, men or women, subjects with or without diabetes, subjects with various stage of CKD, subjects with or without CAD, subjects with HFrEF or HFpEF, and subjects with decompensated or de novo HF, after accounting for age and sex. (Fig. 2) There were significant interactions of RL in the prediction of mortality between subjects with or without CAD and with HFrEF or HFpEF. In other words, RL was more likely to predict mortality in subjects with CAD than without CAD, and in subjects with HFrEF than with HFpEF.

### The prognostic impacts of hemographic indices was abated along the follow up

**Figure 3:**
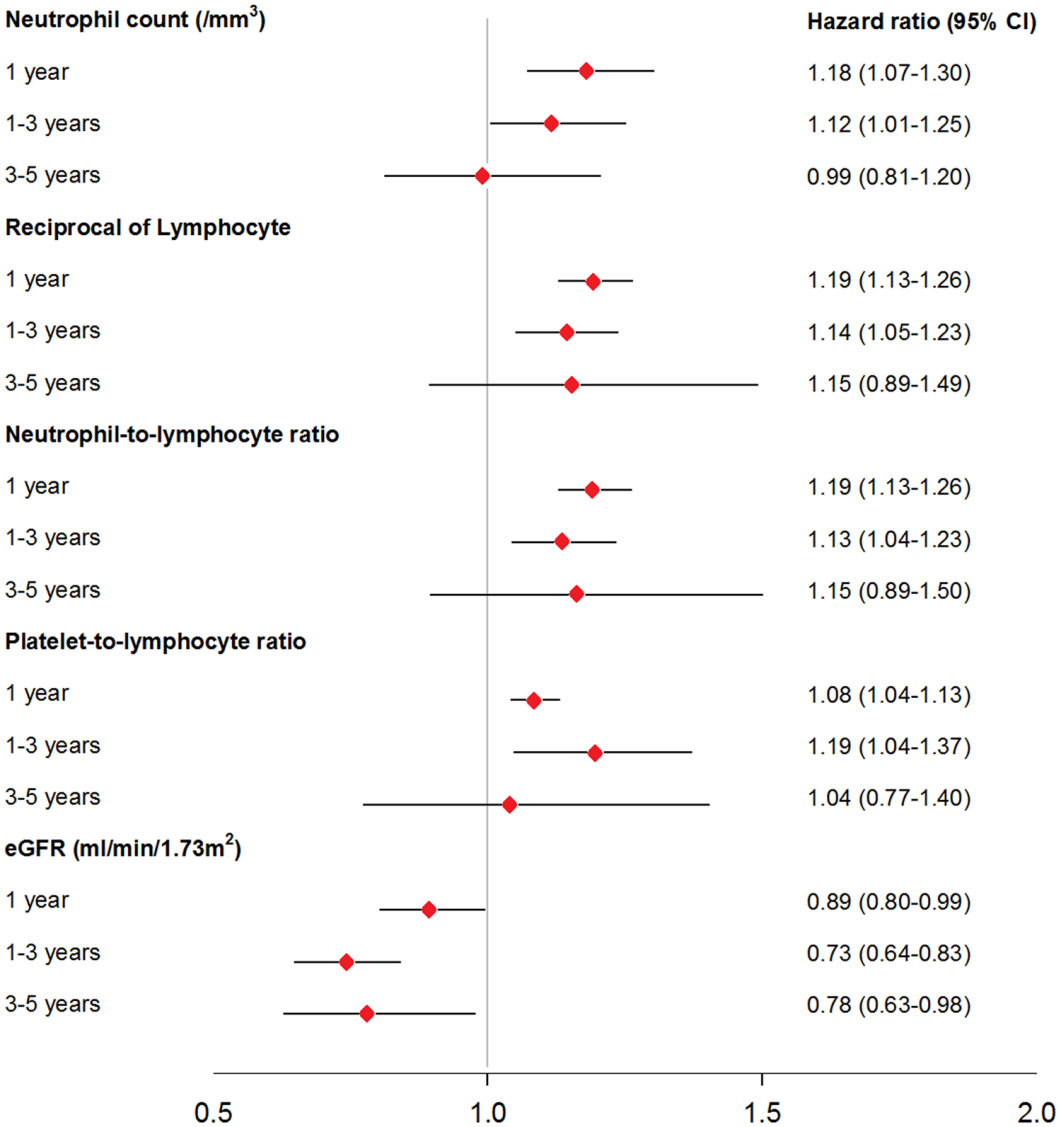
Hazard ratio (HR) and 95% confidence interval (CI) of per-1SD increase of neutrophil count with log transformation, reciprocal of Lymphocyte count, neutrophil-to-lymphocyte ratio, platelet-to-lymphocyte ratio, and estimated glomerular filtration rate (eGFR) for mortality in specific follow-up periods, after accounting for age and sex.

The variety of the predictive values of hemographic indices to mortality was demonstrated in Fig. 3. The patient number at risks was 1923 within 1 year, 1508 between 1 to 3 years, and 1166 between 3 to 5 years, respectively. After accounting for age and sex, the hemographic indices were correlated with mortality within the first year of follow-up. Conditioned on the survivals, Cox regression analysis showed that neutrophil count, RL, NLR and PLR were associated with mortality between 1 to 3 years of follow-up period, independent of age and sex. Beyond 3 years follow-up, none of the hemographic indices was independently related to mortality among the survivors. However, eGFR was consistently correlated with mortality in every follow-up period. The incidence rates of mortality per 100 person-year were demonstrated in Table 3, stratified by the tertiles of RL and the follow-up time slots. In the first year and between 1 to 3 years of follow-up, the incidence rates significantly increased along with the tertiles distributions of RL (both crude P value of the trend < 0.01). In contrast, the incidence rates did not augment together with increasing RL between 3 to 5 years of follow-up. After adjustment of age, gender, mean blood pressure, LVEF, hemoglobin, eGFR, sodium, and prescribed medications, the mortality rate significantly raised along with RL only in the first year. (Table 3).

**Table 3:** The associations of mortality and reciprocal of lymphocytes during the follow-up time slots.

	**Incidence rate (per 100 person-year)**	**Crude RR (95% RR)**	**P for trend**	*Adjusted RR (95% CI)	**P for trend**
**Within 1 year**					
*RL tertiles 1*	17.2	1	<**0.01**	1	<**0.01**
*RL tertiles 2*	21.2	1.23 (0.94–1.62)		1.02 (0.72–1.44)	
*RL tertiles 3*	39.5	2.29 (1.80–2.93)		2.01 (1.47–2.73)	
**1–3 years**					
*RL tertiles 1*	11.2	1	<**0.01**	1	**0.07**
*RL tertiles 2*	19.3	1.72 (1.30–2.28)		1.51 (1.09–2.11)	
*RL tertiles 3*	21.4	1.91 (1.44–2.52)		1.40 (1.00–1.96)	
**3–5 years**					
*RL tertiles 1*	8.6	1	**0.07**	1	**0.58**
*RL tertiles 2*	15.4	1.78 (1.15–2.78)		1.30 (0.75–2.26)	
*RL tertiles 3*	13.1	1.53 (0.95–2.44)		0.84 (0.46–1.55)	

RL: reciprocal of lymphocytes; RL tertiles 1 was with the lowest RL levels; CI: confidence interval; RR: rate ratio; *Adjust for age, sex, mean blood pressure, LVEF, hemoglobin, eGFR, sodium, and prescribed medications.

## Discussion

The major findings of this study include the following: (1) hemographic indices were independent predictors of mortality in patients hospitalized for AHF; (2) among the hemographic indices, RL might be the most valuable index related to long-term outcomes of AHF with either reduced or preserved LVEF; (3) as dominant markers of acute inflammation, hemographic indices were especially useful in the risk discrimination of short and medium-term mortality after heart failure hospitalization. Since hemogram is one of the routine tests in daily practices, the results of this study may support the clinical uses of hemographic indices in the risk reclassifications of patients hospitalized for AHF.

### Inflammation, hemographic indices and heart failure

Systemic inflammation and the related cytokines in heart failure have been associated with the migration of monocytes to the myocardium and the consequent interstitial fibrosis and ventricular remodeling^21^. Numerous studies have demonstrated biomarkers of systemic inflammation, including pro-inflammatory cytokines, C-reactive protein, and erythrocyte sedimentation rate were correlated with the pathogenesis and clinical outcome of heart failure^11,22–24^. It has been known the hemogram varies in parallel with the inflammation, while the white blood cells may further regulate the circuits in the innate and adaptive immune systems^2,25,26^. Furthermore, high NLR and RL were related to elevated jugular vein pressure, increased heart rate, and increased serum B-type natriuretic peptide levels in patient with CHF^13,27^.

### Hemographic indices are risk markers of acute heart failure

In an epidemiological study of 16940 men, Engström *et al*. found white blood cell counts were associated with incidence of heart failure hospitalization over a 23-year follow-up period^28^. Cooper *et al*. further demonstrated an elevated white blood cell count of > 7,000 was significantly correlated with mortality in patient with ischemic heart failure^29^. Moreover, both neutrophil and lymphocyte have been strongly and independently related to heart failure hospitalization, survival, and survival free from heart transplantation in patients with acute myocardial infarction or advanced heart failure^30–32^. NLR, a marker combining the two lineages of white blood cells, demonstrated better prognostic values of in-hospital mortality and post-discharge outcomes in patients with AHF^13,33^. The superiority of PLR in predicting immediate effects of revascularization and clinical outcomes in subjects with acute myocardial infarction has also been addressed^7,34,35^. In the present study, all the hemographic indices at the initial presentation were associated with long-term survival in patients hospitalized for AHF, independent of conventional risk factors and prescribed medications. With further adjustments for NT-proBNP, neutrophil count, RL, NLR, and PLR remained correlated with mortality. In addition to extending the use of RL as a prognostic factor in AHF, the results may further support the use of lymphocyte as an index for better risk stratification in patients with AHF, which was in agreement with Seattle heart failure model in CHF^32^.

### Hemographic indices and various subpopulations

The mean values of WBC and NC were in fact within the reference ranges when the reference ranges of WBC and NC are 4500–11000/mm^3^, and 2500–7500/mm^3^ ^36^. However, NLR, PLR and RL values were higher than the healthy population when Erdal Durmus *et al*. have suggested reference ranges of NLR and PLR were 2.5 ± 1.7 and 140 ± 57 in patients without heart diseases^37^.

Majority of the published studies have demonstrated the hemographic indices were independently associated clinical outcomes in patients with HFrEF^14,29,31,32^. Few have shown the prognostic impacts of hemographic indices in patients with HFpEF, not mention in the hospitalized populations. Muthiah *et al*. have investigated in a total of 4133 subjects hospitalization for HFrEF, and they clarified that the lower relative lymphocyte counts was associated with all-cause mortality, cardiovascular death and re-hospitalization for heart failure in the first 100 days after discharge^38^. In this study, we further demonstrated that RL was consistently an independent risk factor for mortality in the patients with either HFrEF or HFpEF. But there was a substantial interaction that RL was especially predictive of long-term survival in patients with HFrEF. The study results might be supported by the data from Sanders-van *et al*. that HFpEF but not HFrEF was already an inflammatory disease^39^. In addition, we also clarified that RL was accordantly related to 5-year mortality in patients ≥ and < 80 years, man and woman, and patients with or without diabetes or various stages of CKD. Again, we reported an authentic interaction of RL and CAD in predicting mortality that per 1-SD increase of RL was associated with additional 46.4% and 13.8% mortality in subjects with and without CAD, respectively. Such findings were in accord with Wang *et al*. that hsCRP was a prognostic factor only in patients with Takayasu arteritis if CAD was presented^40^. The study results may support that inflammation was a significant risk factor for mortality in AHF with various characteristics, especially in the subjects with CAD and reduced LVEF.

### Eminent performance of hemographic indices in predicting short and medium-term mortality in acute heart failure

Conditioned on the survivals, we have demonstrated in this study that hemographic indices could predict mortality in AHF outstandingly within the first year after index hospitalization. Comparing to renal function that eGFR was independently associated with mortality within every time slot of within 1 year, and between 1–3 years and 3–5 years. The study results might suggest the inflammation indexed by hemographic indices was particularly essential to recognize subjects at risk of incurring adverse events soon after. There was therefore an unmet need to develop tailored therapeutic strategy for the high-risk population, tagged with high hemographic indices.

### Study Limitations

Indeed, there were several limitations of this study. First, the mean age of the study population was 76.3 ± 12.6 years, which might be the oldest population, reported with hemographic indices. There would be concern to generalize the study results to the younger populations. However, we have shown no statistical interaction with the prognostic values of the hemographic indices and various age populations. Furthermore, given the nature of a registry study, there would still be selection bias even we have adjusted all the confounders to demonstrate the independence of hemographic indices related to mortality. Third, NT-proBNP was only available in 33% of the study population. But in the 646 patients with available NT-proBNP data, there were 196 mortalities. We therefore still have sufficient power to demonstrate hemographic indices were related to outcomes, independent of NT-proBNP. Forth, we did not measure any novel inflammatory marker, such as interleukin-6, tumor necrosis factor-alpha, and high sensitivity C-reactive protein in this study to compare their prognostic impacts with hemographic indices. However, all the hemographic indices significantly correlated with C-reactive protein (CRP) among a total of 900 patients with available data in this study. In addition, hemographic indeices remained predictive of 5-years mortality (data not shown) when CRP was further accounted for. The results may suggest the hemographic indices were not only the reflection of systemic inflammation. Fifth, we did not obtain hemographic indices at discharge. Whether the fluctuations of hemographic indices during hospitalizations were predictive of clinical outcomes remained elucidated. Lastly, the study was lack of incident morbidities, such as re-hospitalization for HF. Further work was needed to address the risks of hemographic indices for mortality and morbidity.

## Conclusion

Inflammation, indexed by hemographic indices were independent risk factors for long-term mortality in patients hospitalized for AHF with either HFeEF or HFpEF, especially when the indices were composed of lymphocyte. However, the prognostic impacts of inflammation in AHF were dismal with time. In other word, hemographic indices were useful to recognize subjects who were at high risks of incurring adverse event soon after the hospitalization for AHF.

## Electronic supplementary material

Table S1
