# Supplementary material for: Hemographic indices are associated with mortality in acute heart failure
Source: Sci Rep. 2017 Dec 19;7:17828. doi: 10.1038/s41598-017-17754-8 (PMC5736628; doi:10.1038/s41598-017-17754-8)
Supplement: Supplementary file 1 — Table S1 [file 41598_2017_17754_MOESM1_ESM.doc]

**Hemographic indices are associated with mortality in acute heart failure**

Wei-Ming Huang^a,c^, Hao-Min Cheng^b,c^, Chi-Jung Huang^b,c^, Chao-Yu Guo ^d^, Dai-Yin Lu^a,c^, Ching-Wei Lee^a,c^, Pai-Feng Hsu^a,c,d^, Wen-Chung Yu^a,c^, Chen-Huan Chen^a,c,d^, Shih-Hsien Sung^a,c,d^

^a^Department of Medicine, and ^b^Department of Medical Education, Taipei Veterans General Hospital, Taipei, Taiwan;

^c^Department of Medicine, and ^d^Department of Public Health, National Yang-Ming University, Taipei, Taiwan

| **Table S1. Predictors of mortality identified by uni-variable Cox regression analysis** | | | |
| --- | --- | --- | --- |
|  |  | **Hazard Ratio** | **P value** |
| Age, 1SD=12.7 years | | 1.326 (1.228-1.433) | <0.001 |
| Sex* | | 1.067 (0.924-1.233) | 0.378 |
| Mean Blood Pressure, 1SD=21.9 mmHg | | 0.849 (0.781-0.922) | <0.001 |
| LVEF, 1SD=20.4 % | | 0.921 (0.866-0.980) | 0.01 |
| WBC count, 1SD=1.44/mm^3^ | | 1.623 (0.876-3.008) | 0.124 |
| Neutrophil count, 1SD=1.60/mm3 | | 2.791 (1.641-4.747) | <0.001 |
| Reciprocal of Lymphocyte, 1SD=6.3 | | 1.162 (1.115-1.211) | <0.001 |
| Neutrophil-to-lymphocyte ratio, 1SD= 6.1 | | 1.160 (1.112-1.210) | <0.001 |
| Platelet-to-lymphocyte ratio, 1SD=189.5 k/mm3 | | 1.090 (1.052-1.129) | <0.001 |
| Hemoglobin, 1SD= 2.25 g/dl | | 0.788 (0.737-0.842) | <0.001 |
| eGFR, 1SD= 30.0 ml/min/1.73 m^2^ | | 0.799 (0.741-0.862) | <0.001 |
| Sodium, 1SD= 4.75 mEq/L | | 0.907 (0.848-0.970) | 0.004 |
| NT-proBNP, 1SD=3.74 pg/ml | | 1.513 (1.303-1.756) | <0.001 |
| RAS blockade† | | 0.648 (0.549-0.764) | <0.001 |
| Beta-blockers† | | 0.648 (0.567-0.741) | <0.001 |
| Spironolactone† | | 0.790 (0.692-0.902) | 0.001 |
| * men versus women; † user versus non-user  eGFR: estimated glomerular filtration rate; LVEF: left ventricular ejection fraction; NT-proBNP: N-terminal pro-brain natriuretic peptide; RAS blockade: renin-angiotensin system blockade; WBC: white blood cell; SD: standard deviation | | | |
